# Evidence of West Nile Virus Circulation in Lebanon

**DOI:** 10.3390/v13060994

**Published:** 2021-05-26

**Authors:** Renée Zakhia, Alan P. Dupuis, Fayçal Khodr, Mahdi Fadel, Laura D. Kramer, Nabil Haddad

**Affiliations:** 1 Laboratory of Immunology and Vector-Borne Diseases, Faculty of Public Health, Lebanese University, Fanar 6573/14, Lebanon; renee.zakhia@gmail.com (R.Z.); faycalkh@hotmail.com (F.K.); mahdi_fadel99@hotmail.com (M.F.); 2 Arbovirus Laboratories, Wadsworth Center, New York State Department of Health, Albany, NY 12159, USA; alan.dupuis@health.ny.gov (A.P.D.II); laura.kramer@health.ny.gov (L.D.K.); 3 Department of Biomedical Sciences, State University of New York at Albany School of Public Health, Albany, NY 12201, USA

**Keywords:** Lebanon, Middle East, West Nile virus, flavivirus, seroprevalence, neutralization

## Abstract

West Nile virus (WNV) has never been reported from Lebanon. Yet, this country is located on the flyway of migratory birds in the Middle East region. Serological screening was conducted to assess the potential circulation of this virus. Human, horse, and chicken sera were collected from the Bekaa and North districts. Specific IgG and IgY were first screened by ELISA. Then, positive samples were confirmed by plaque reduction neutralization test (PRNT). Besides this, adult mosquitoes were collected and tested for the presence of WNV RNA using conventional RT-PCR. Sera screening revealed a seroprevalence rate reaching 1.86% among humans and 2.47% among horses. Cross-reactions revealed by ELISA suggested the circulation of flaviviruses other than WNV. None of the tested mosquitoes was positive for WNV. The observed results constitute strong evidence of local exposure of the Lebanese population to this virus and the first report of equine WNV in Lebanon.

## 1. Introduction

West Nile virus (WNV; *Flavivirus*; *Flaviviridae*) is a widespread mosquito-borne virus. It is transmitted to vertebrates by the bite of a broad range of mosquito species, mainly members of the *Culex* genus. Birds are its natural host. When infected, they often remain asymptomatic except in the United States where significant mortality is observed [1]. Other vertebrates, such as humans and horses, are considered dead-end hosts. In humans, the infection is asymptomatic in 80% of cases. Otherwise, infected individuals develop an acute febrile illness. Severity varies from a self-limited infection to encephalitis causing long-term disability and, in 10% of neurologic cases, death.

West Nile fever (WNF) is widespread throughout Africa, parts of Europe, the Middle East, West Asia, and Australia. Following its introduction in 1999 to the United States of America, WNV succeeded in establishing itself in several countries in the Western Hemisphere [2]. Outbreaks have occurred recurrently in several countries, especially in those on the major migratory routes of birds, such as Greece, Italy, and Russia. The reported numbers of cases vary yearly with significant differences between countries. In Europe, for example, in 2018, ECDC reported 1605 cases, of which 77% originated from Italy, Greece, and Romania. The infection rate in that year was eight times higher than in 2017 [3].

The Middle East region is endemic for this pathogen. West Nile fever cases are regularly reported from Israel [4,5] and Turkey [6]. Serological evidence of WNV circulation has been documented in several countries in the region, including Jordan [7] and Egypt [8]. While WNF has never been reported in Lebanon, we hypothesize that this arbovirus circulates in the country. Indeed, Lebanon, like its neighbouring countries, is on the flyways of migrating birds, the principal reservoir of the virus. The present study aims to assess the circulation of WNV in Lebanon. To that end, we screened human, horse, and chicken sera in selected geographic locations for WNV-specific antibodies and we tested local *Culex* populations for the presence of WNV RNA.

## 2. Materials and Methods

### 2.1. Blood Collection

**Figure 1:**
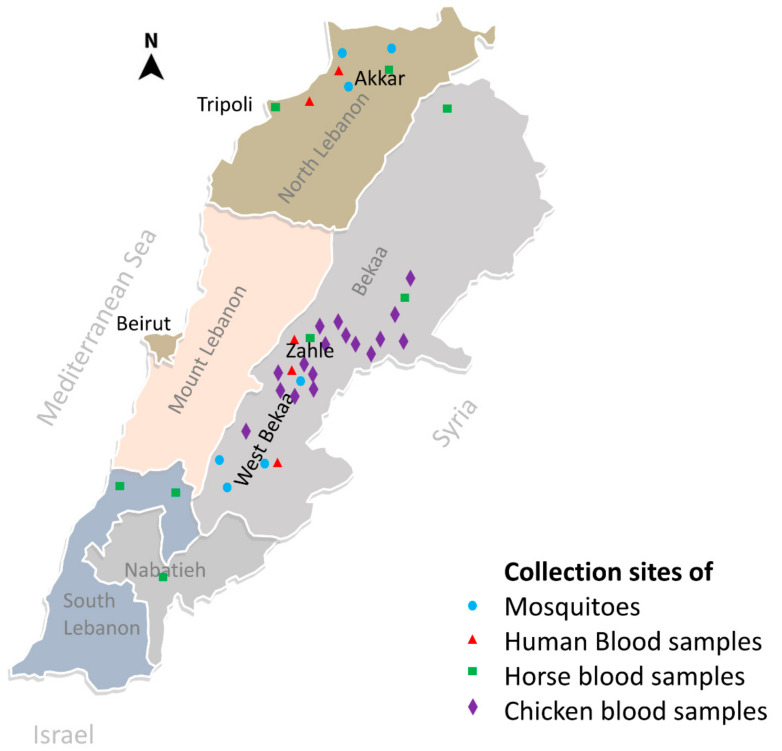
Map of Lebanon indicating the collection sites of mosquitoes and blood from human, horse, and chicken hosts.

The serological study involved three types of hosts: humans, horses, and chickens. Two populations were included for the serosurvey in humans. The first population consisted of residents of two adjacent districts in the Bekaa valley: West-Bekaa (130,000 inhabitants, 425 Km^2^) and Zahle (320,000 inhabitants, 425 Km^2^) (Figure 1). This agricultural region is characterized by the presence of large water systems used as a stopover by migratory birds. The second population was from Akkar district (420,000 inhabitants, 788 Km^2^), north of Tripoli, a relatively underdeveloped region with considerably important farming activities. All involved participants were recruited during summer (June to September) 2013 among outpatients referred to laboratories of hospitals (three in the Bekaa region) and dispensaries (two in Akkar) for blood testing. Each consenting participant donated 5 mL of blood and filled out a questionnaire that included information on his/her travel history.

Equine blood was collected from June to October 2015 from farms and riding clubs in rural areas in several districts of the Bekaa region (including West Bekaa and Zahle), North region (including Akkar), and South regions (Figure 1). Information on each horse (breed, geographic origin, age, vaccine history) was obtained from the owner or using a microchip reader when possible.

Blood was also collected from chickens raised in the backyards of villagers in the Bekaa region, including the West Bekaa district.

The minimal sample size calculation was conducted on EpiInfo software (version 7.2.2.6) using the following parameters: an expected seroprevalence of 8% based on what is observed in neighbouring countries [7], an acceptable error margin of 4%, and an alpha value of 5%. The computed minimal sample size is 169 for horses and 177 for humans for each study region.

### 2.2. Screening of Sera by Enzyme-Linked Immuno-Assay

Sera obtained from collected blood of different hosts were tested for the presence of specific antibodies (IgG and IgY) using an in-house indirect enzyme-linked immunosorbent assay (ELISA) as described by Ebel and collaborators (2002) [9]. Two types of antigens were prepared: a positive antigen using Vero cells infected with WNV (3100365 strain ref, isolate collected from a pool of *Culex* species mosquitoes, Staten Island, NY, 2000) and a negative antigen with uninfected cells as described [10]. The optimal dilutions of both antigens and the secondary antibodies were decided using confirmed positive and negative control sera for each host. Nunc-Immuno MicroWell ELISA plates of 96 wells (Sigma, St. Louis, MO, USA) were coated using 50 µL of antigens diluted to 1:200 in coating buffer (0.015 M Na_2_CO_3_, 0.035 M NaHCO_3_, pH 9.6). The negative antigen was placed in each well of every third column (1st, 4th, 7th, and 10th) while the positive antigen was distributed in the wells of the remaining columns of each plate. The antigens were allowed to bind during an overnight incubation at 4 °C. The following day, the coat solution was discarded, plates washed three times with Phosphate Buffered Saline with 0.05% Tween (PBS-T), then 100 μL blocking buffer (5% skimmed milk in PBS-T) was added, and plates were left in a humid chamber at 37 °C for 1 h. Afterward, the blocking solution was discarded and plates were washed as above. A volume of 50 μL of each serum, diluted 1:200 in PBS-T with 0.5% Bovine Serum Albumin (BSA), was placed to one negative and two positive antigen-containing wells. Plates were then incubated in a humid chamber and left for 1 h at 37 °C. After washing, 50 μL of goat anti-human IgG (H+L) horseradish peroxidase (HRP) secondary antibody (Southern Biotech, Birmingham, AL, USA), diluted 1:4000 in PBS-T-BSA buffer, were added to each well. For horse and chicken sera, goat anti-equine IgG (H+L) HRP (Southern Biotech, USA) or goat anti-chicken IgY (H+L) HRP (Southern Biotech, USA) were respectively used at the same dilution. After incubation and washing as above, potentially formed immune complexes were revealed by the addition of 50 μL of 3,3′,5,5′-Tetramethylbenzidine (TMB) substrate (Eurobio, Les Ulis, France) for 10 min. The reactions were stopped with 50 μL of 1:20 HCl, and the optical density (OD) of each well was read at 450 nm. For each plate blank (wells without test sera), controls (positive and negative) were included. For each serum, a ratio of the mean of OD obtained on the positive antigen (ODP) over the OD obtained on the negative antigen (ODN) was calculated. Human sera, for which OPD/ODN >2 were considered positive, were tested by seroneutralization for confirmation. For horses and chicken sera, in order to increase the sensitivity, those with ODP/ODN >1.20 were tested by seroneutralization.

### 2.3. Confirmation by Plaque Reduction Neutralization Test

All sera revealed positive by ELISA (at the fixed threshold) were tested by PRNT as previously described [9]. Vero cell suspension (2.0 × 10^5^ cells/mL) was prepared and dispensed (3 mL/well) in 6 well plates which were then incubated at 37 °C and 5% CO_2_. Test sera were serially diluted from 1:5 through 1:160 in BA-1 diluent (Medium-199H, 0.05 M Tris pH 7.6, 1% bovine serum albumin, 0.35 g/L sodium bicarbonate, 100 units/mL penicillin, 100 µg/mL streptomycin, and 1 µg/mL fungizone) to 100 µL. They were then mixed to 100 µL of WNV suspension (Strain 3100365) containing 200 PFU and incubated overnight at 4 °C. The following day, 100 µL of each serum-virus mixture was added onto confluent monolayers of the prepared Vero cell plates and allowed to adsorb at 37 °C for 1 h, after which an agarose overlay (3 mL/well) was added, and incubation was continued. When virus plaques became visible, a second overlay of nutrient-agar (3 mL/well) containing neutral red (1mL of neutral red/100 mL of overlay) was added. Plates were then returned to the incubators. Plaques were counted on the 3rd day after the viral inoculation. The titer was determined as the inverse of the highest dilution of serum neutralizing 90% of the inoculum when compared to the control (back-titration).

In order to assess the potential cross-reactivity with other flaviviruses, a set of WNV ELISA-positive sera were tested for DENV by PRNT following the same protocol used for WNV. DENV-2 serotype (New Guinea C) was used in this assay.

### 2.4. Screening for WNV in Mosquitoes

#### 2.4.1. Mosquito Collection and Identification

Adult mosquito specimens were collected around houses and in farms between August and September 2014 from seven locations: 4 in West Bekaa and 3 in Akkar districts (Figure 1). Two types of traps were used: CDC (Centres for Disease Control) light trap (John W. Hock, Gainsville, FL, USA) fitted with a CO_2_ source and BG-sentinel traps (Biogents ^®^) with Bg-lure attractant. Collected mosquitoes were identified using regional keys [11,12,13] and then pooled (up to 50 specimens) in RNA stabilization solution (MCLAB, San Francisco, CA, USA) and conserved at −80 °C for subsequent molecular analysis.

#### 2.4.2. Screening for WNV

Total RNA was extracted from mosquito pools using NucleoSpin RNA isolation kit (Macherey-Nagel, Dueren, Germany). To produce cDNA, standard reverse transcription polymerase chain reaction (RT-PCR) using GeneAmp Gold RNA PCR Reagent Kit (Applied Biosystems, Foster City, CA, USA) was performed as per the manufacturer’s protocol. Subsequently, a 326 base-pairs fragment of the NS5 gene was amplified. The PCR mix included 25 µL of PCR Master Mix (Thermo Scientific, Waltham, MA, USA), WN 9794 (GGA-ACC-TGC-TGC-CAA-TCA-TAC-CAT-C), and WNV NS5 WN 9483 (CAC-CTA-CGC-CCT-AAA-CAC-TTT-CAC-C) [14,15] primers at a final concentration of 0.5 µM, 3 µL cDNA, Q.S of nuclease-free water for a final volume of 50µL. PCR cycling parameters were as follows: Enzyme activation at 95 °C for 10 min, PCR (denaturation, annealing, extension) with 35 cycles at 94 °C for 45 s, 56 °C for 45 s, 72 °C for 1 min, PCR final step (elongation) at 72 °C for 10 min. The obtained amplicons were run on a 1.5% agarose gel electrophoresis and revealed using gel red nucleic acid gel stain (Biotium, Fermont, CA, USA).

## 3. Results

A total of 1091 serum samples were analysed: 803 from the Bekaa area and 288 from Akkar. Participants’ age ranged from 9 to 85 years (Median 39) with the highest representation of those aged between 21 and 30 years old. The majority of participants (63. 34%) were females.

**Figure 2:**
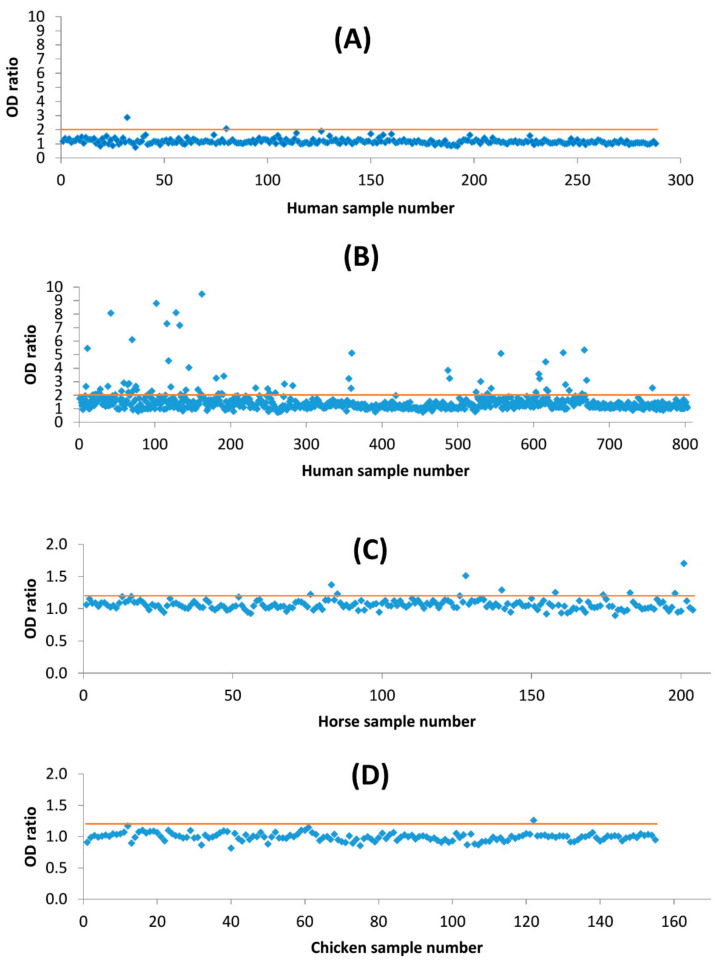
Optical density ratios (ODP/ODN) obtained by ELISA: (**A**) for human sera from the West Bekaa and Zahle districts, (**B**) from Akkar district, (**C**) of horse sera, and (**D**) of chicken sera from the Bekaa region. The red line represents the considered threshold (OD ratio = 2 for human sera; OD ratio = 1.2 for animal sera).

Sera analysis by in-house ELISA showed that 69 individuals from the Bekaa (8.62%, *n* = 803) and 2 from North area (0.69%, *n* = 288) had ODP/ODN > 2 and therefore were considered tentatively seropositive for WNV (Figure 2A,B). Of those, only 17 had WNV-specific neutralizing antibodies detectable at a titre of 10 or higher: 15 (1.86%) from the Bekaa and 2 (0.69%) from the North region. The highest antibody titres were markedly observed among sera of patients older than 65 years old. Eight participants had never travelled out of Lebanon (Table 1). Besides this, for those with travel history to endemic countries such as the United States, exposure to WNV in the destination countries could not be determined based on the limitations of the questionnaire. Therefore, autochthonous exposure to this virus is not excluded for those patients.

Thirty sera that had OD ≥2.5 were tested for DENV by PRNT. Twenty of them had no neutralizing antibodies neither for WNV nor for DENV. Eight of the remaining 10 sera had titres higher for WNV than for DENV (Table 1), which consolidate their identity. Nevertheless, two sera (one belonging to a patient with no travel history) had at least fourfold greater titres of neutralizing antibodies to DENV than to WNV and therefore cannot be considered positive for WNV.

According to the Lebanese ministry of agriculture, there are around 4000 horses in Lebanon. Blood samples were taken from 202 horses from 16 farms in the country (Figure 1). The majority were females (69.31%) and belonged to a local breed called Alef (70.56%). Horses’ age ranged from 4 months to 22 years with an average of 8.74 years. ELISA testing showed that 10 sera (4.95%) samples had an OD ratio above 1.2 threshold (Figure 2). Of those, five (2.47%) had neutralizing antibodies for WNV at titres of 10 or higher (Table 2). All of them were of Alef breed from Tripoli, Akkar in the North, and from Nabatieh, South of the country. None of the animals had a history of cross-border movement but evidence of circulation between different Lebanese districts. In addition, sera of 163 chickens from the Bekaa were tested. Only one had an OD ratio higher than 1.2 with no detected neutralizing antibodies.

Mosquito capture during 166 trap-days in 7 localities in West Bekaa and Akkar districts (Figure 1) yielded 6166 adult specimens. Subsequent morphological identification showed that 96.83% belonged to *Culex pipiens* species. The remaining specimens (3.17%) belonged to four other species: *Culex martini, Culex perexiguus, Culiseta longiareolata*, and *Orthopodomyia pulcripalpis* (Table 3). Total RNA was extracted from pooled specimens from both districts. They all screened negative for WNV NS5 gene.

## 4. Discussion

Lebanon is considered non-endemic and non-enzootic for WNV. To date, no WNF cases have been officially reported. The detection of relatively high titres of neutralizing antibodies IgG (≥320) in participants who had never travelled out of the country confirms the local circulation of this virus. Potential cases of West Nile Fever (WNF) may go unnoticed in the country probably because infectious disease specialists are not familiar with this arboviral agent. Consequently, a West Nile infection probably would be clinically classified as an unexplained viral infection. The majority of seropositive cases were from the Southern part of the Bekaa region, a rural area with several water plans (Aammiq swamps and Qaraoun lake) and stopovers for migratory birds, which makes it suitable for the introduction and local spread of WNV. This area is the geological extension of the Great Rift Valley in Israel where a cross-sectional study, involving more than 3100 healthy individuals, showed that it had the highest seroprevalence (13.5%) of WNV (with evidence of seroneutralization) of the country [16]. In this bordering country, WNV has been responsible for several outbreaks. In the years 2000 and 2015, hundreds of cases were reported, mainly from the central and northern regions close to Lebanon [4,5].

Unexpectedly, the observed seroprevalence rate, especially in the Bekaa region, is comparable to that reported from endemic areas such as New York (2.6%) [17] and northern Italy (2.08%) [18]. Closer to Lebanon, in Greece, serosurveys showed a positive rate of 1.5% (95% C.I.: 1.2–2.0) following confirmation by PRNT [19]. In the neighbouring Turkey, WNV seroprevalence rates also determined by PRNT were much higher reaching, 14.9% in Mersin Southeast Turkey [20]. In Jordan (Hashimiah region) and Iran (Mashhad region), which, like Lebanon, are considered non-endemic for WNV, the seroprevalence rates were estimated at 8% and 11%, respectively, following screening by ELISA [7,21]. The observed rate in Lebanon is probably overestimated as suggested after assessment of cross-reactivity with DENV. Sera with very low titres (10) of neutralizing antibodies should be cautiously interpreted.

In Lebanon, in a previous serosurvey conducted by Gallian and collaborators (2010) among 639 blood donors, 6 were WNV seropositive by ELISA, among which only 3 (0.47%) had neutralizing antibodies positive by PRNT at titres higher than 1/10. Seropositive participants lived in South Lebanon, Beirut, and the Bekaa; however, the authors did not discuss their travel history and whether they were locally exposed to the virus [22].

The detection of seropositive horses among local breeds is additional evidence of WNV circulation in Lebanon. It is the first record of equine exposure to WNV in Lebanon. According to the information system of the OIE (Office International des Epizooties), no West Nile infection has been reported in animals since 2006 not only in Lebanon but also in Syria [23], while cases in horses have been reported from Israel and Jordan [24,25]. The observed seroprevalence (2.47%) remains significantly lower than that reported in endemic countries of the region where the seroprevalence after neutralization was as high as 21% in Turkey [26], and 48.6% and 84.6% in Palestine and Israel, respectively [24]. Nevertheless, the measured seroprevalence is comparable to that reported from Iran between 2010 and 2012 where 2.8% (*n* = 315) were seropositive by ELISA [27].

The absence of WNV-specific antibodies (IgY) among poultry samples from the Bekaa suggests that there was no obvious active circulation of this virus at the time of the study.

Interestingly, 52 human sera were seropositive by ELISA, some of them with high OD ratio, but did not have neutralizing antibodies for WNV. This suggests the circulation in the study region of other flaviviruses closely related to WNV, such as Zika virus (ZIKV), Usutu virus (USUV), and Dengue virus (DENV). To our knowledge, the first two viruses have never been reported in Lebanon. ZIKV had not spread yet at the time of sera collection in 2013. The only report of USUV from the Middle East is from Israel where the virus was detected in mosquito pools collected from Haifa (North), and neutralizing antibodies were detected in 10.8% of horses, predominantly from the northern part of the country [28,29]. Recent studies, including an examination of zoo specimens collected in France and blood donors from Italy, revealed little to no cross-reactivity in neutralization assays between WNV and USUV, especially in samples with high PRNT90 titers (>80) [30]. Since USUV was not included in this study, it is impossible to assess cross-reactivity in the human and horse samples with low neutralization titers to WNV and DENV. Future studies should include USUV to assess the transmission of flaviviruses in the Middle East. On the other hand, Dengue fever (DENF) was endemic in Lebanon before 1970; more than 100,000 cases were reported during 1945–1946 [31]. This disease was eliminated from the country after the implementation of the malaria eradication program that led also to the elimination of *Aedes aegypti*, the only vector of the virus at that time. In 2003, the tiger mosquito (*Aedes albopictus*), a secondary vector of DENV, was observed for the first time in Lebanon [32]. Local strains of this mosquito demonstrated a relatively low capacity to transmit DENV under experimental conditions [33]. In 2012, a patient with no prior travel history was diagnosed with Dengue fever [34]. The detection in two sera (one belonging to a patient with no travel history) of at least four-fold greater titres of neutralizing antibodies to DENV-2 than to WNV is suggestive of local circulation of DENV or eventually other flavivirues, which warrants further investigation.

Entomological data showed a high presence of *Culex pipiens*, the known primary vector of WNV. A previous experimental study on Lebanese strains of this mosquito demonstrated a high capacity to transmit WNV [35]. Moreover, another potential vector, *Culex perexiguus*, was also present but in limited numbers. This species favors enzootic (bird to bird) and epizootic (bird to horse) transmission of WNV [36]. In Israel, analysis of a large mosquito collection allowed the detection of WNV in 2.39% and 9.22% of *Cx. pipiens* and *Cx. perexiguus* pools, respectively [37]. The absence of WNV in the tested mosquitoes in Lebanon is suggestive of low circulation of the virus at the time of the collection. Therefore, higher numbers of mosquitoes need to be tested, preferably with a TaqMan reverse transcriptase PCR assay that is more sensitive than RT-PCR [15].

In conclusion, during this study, we put forward strong evidence of humans’ and horses’ exposure to WNV in Lebanon. The virus seems more prevalent, at least among humans, in the Bekaa region than in Akkar. Serological data revealed exposure to other flaviviruses including DENV. Further studies are needed to determine the identity and the extent of the spread of these viruses.

## Figures and Tables

**Table 1:** List of seropositive humans that had WNV- and DENV-neutralizing antibodies by PRNT at titres ≥ 10.

Districts	Serum	WNV		DENV	Travel History	Age (Years)	Gender
		OD Ratio (ELISA)	Titre (PRNT90 *)	Titre (PRNT90 *)			
West Bekaa and Zahle	**B9**	**2.64**	**40**	Neg	No	75	Female
	B11	5.47	≥320	40	No	70	Female
	B42	8.08	≥320 **	20	Canada	83	Male
	B116	7.30	≥320	20	No	80	Male
	B118	3.24	10	≥40 **	No	43	Male
	B121	2.61	10	≥40	Venezuela and Syria	51	Female
	B133	5.00	160	Neg	Egypt	31	Male
	B162	5.22	≥320	Neg	No	65	Female
	12	2.83	≥320	ND	No	85	Male
	23	2.71	10	ND	Venezuela	34	Male
	99	3.23	10	ND	No	58	Female
	235	3.85	160	ND	Brazil and Syria	66	Female
	237	3.25	160	ND	Syria	73	Male
	T57	5.08	≥320	Neg	USA	76	Female
	T167	5.35	≥320	Neg	USA	82	Male
Akkar	U91	2.08	20	ND	Germany	67	Female
	U284	2.86	20	ND	No	81	Male

* PRNT90 the inverse of the highest serum dilution that neutralizes 90% of the viral inoculum. ** The highest tested dilution is 1/320 for WNV and 1/40 for DENV. ND, not determined; Neg, negative.

**Table 2:** List of seropositive horses that had neutralizing antibodies by PRNT at titers ≥ 10.

Serum #	Titre (PRNT90 *)	OD Ratio (ELISA)	Age (Years)	Sex **	District/Region
83	160	1.371	7	Male	Tripoli/North
128	160	1.511	7	Female	Akkar/North
183	10	1.246	20	Female	Nabatieh/South
199	40	1.238	10	Female	Nabatieh/South
202	320	1.701	7	Female	Nabatieh/South

* PRNT90 the inverse of the highest dilution that neutralizes 90% of the viral inoculum. ** All horses were of Alef (local) breed.

**Table 3:** Species composition of collected mosquitoes in West Bekaa and Akkar districts during summer 2014.

District	West Bekaa			Akkar			Total	
Nb of Trap-Days	78			88				
	**Male**	**Female**	**Total**	**Male**	**Female**	**Total**	**Nb**	**%**
*Culex pipiens*	440	2249	2689	511	2771	3282	5971	96.83
*Culex perexiguus*	0	10	10	0	82	82	92	1.49
*Culex martinii*	2	30	32	0	9	9	41	0.67
*Culiseta longiareolata*	19	17	36	0	12	12	48	0.78
*Orthopodomyia pulcripalpis*	12	2	14	0	0	0	14	0.23
Total	473	2308	2781	511	2864	3385	6166	

## Data Availability

The data presented in this study are contained within the article.
